# Supervisor leadership in relation to resident job satisfaction

**DOI:** 10.1186/s12909-016-0688-z

**Published:** 2016-08-01

**Authors:** Martha A. van der Wal, Johanna Schönrock-Adema, Fedde Scheele, Nienke R. Schripsema, A. Debbie C. Jaarsma, Janke Cohen-Schotanus

**Affiliations:** 1 Center for Education Development and Research in Health Professions (CEDAR), University of Groningen and University Medical Center Groningen, Groningen, The Netherlands; 2 St. Lucas Andreas Hospital (SLAZ), Amsterdam, The Netherlands; 3 UMCG, FC40 CEDAR, Antonius Deusinglaan 1, 9713 AV Groningen, The Netherlands

**Keywords:** Leadership development, Residency, Postgraduate medical education, Work-based learning

## Abstract

**Background:**

Research from outside the medical field shows that leadership behaviours influence job satisfaction. Whether the same is true for the medical training setting needs to be explored. The aim of this study was to investigate the influence of residents’ overall appreciation of their supervisor’s leadership and observation of specific supervisor leadership behaviours on job satisfaction.

**Methods:**

We invited residents (*N* = 117) to rate how often they observed certain task and relation-oriented leadership behaviours in their supervisor and overall appreciation of their supervisor’s leadership. Furthermore, they rated their satisfaction with 13 different aspects of their jobs on a 10-point scale. Using exploratory factor analysis we identified four factors covering different types of job satisfaction aspects: personal growth, autonomy, affective, and instrumental job satisfaction aspects. Influence of overall appreciation for supervisor leadership and observation of certain leadership behaviours on these job satisfaction factors were analysed using multiple regression analyses.

**Results:**

The affective aspects of job satisfaction were positively influenced by overall appreciation of leadership (B = 0.792, *p* = 0.017), observation of *specific instructions* (B = 0.972, *p* = 0.008) and *two*-*way communication* (B = 1.376, *p* = 0.008) and negatively by *mutual decision*-*making* (B = −1.285, *p* = 0.007). No effects were found for the other three factors of job satisfaction.

**Conclusions:**

We recommend that supervisors become more aware of whether and how their behaviours influence residents’ job satisfaction. Especially providing *specific instructions* and using *two*-*way communication* seem important to help residents deal with their insecurities and to offer them support.

## Background

Research from outside the medical field shows that leadership behaviours of managers influence employees’ job satisfaction [1]. Whether this finding also applies to the medical *training* setting remains unclear. It is important to unravel the relations between supervisors’ leadership and residents’ job satisfaction because job satisfaction may help to counteract the stress a lot of residents experience [2]. We investigated how residents’ job satisfaction is influenced by their appreciation of their supervisors’ leadership in general and specific leadership behaviours.

Job satisfaction has been defined as “a pleasurable emotional state resulting from the appraisal of one’s job…” [3]. This pleasurable emotional state can be related to several aspects of a job, which means that job satisfaction can be considered a multifaceted concept [4, 5]. Which specific aspects are important for job satisfaction may differ per job type. For physicians specifically several factors have been identified that influence their job satisfaction. Examples are opportunities for personal development, professional accomplishments, control over work planning and content, their relationship with colleagues, management and other medical staff, income, work-life balance and appreciation from patients [5–8].

From the organizational literature we know that employees’ job satisfaction is positively influenced by overall appreciation of their managers [9]. Furthermore, specific leadership behaviours such as coordinating and structuring but also how supervisors communicate have been found to influence employee job satisfaction [10, 11]. According to the Situational Leadership Theory (SLT) of Hersey and Blanchard [12], it depends on the work situation and experience level of the employee which specific leadership behaviours are beneficial for job satisfaction. Leadership behaviours can vary over two domains according to the SLT: task-oriented and relation oriented leadership behaviours. Task-oriented leadership behaviours are instructive and directive. Such behaviours are considered appropriate for unstructured and unclear situations in which employees feel uncertain, insecure and are unable to function autonomously. Relation-oriented leadership behaviours refer to providing support to the employee like establishing and maintaining good communication and social support. Such behaviours are considered appropriate for transparent work contexts and situations where employees are capable of functioning autonomously.

We wondered whether the findings reported in organizational literature also apply to a residency training setting and analyzed the relation between supervisor leadership behaviour and resident job satisfaction. Residents in a clinical training context may even be more dependent on their supervisors than employees in a manager-employee setting, because they are also dependent on their supervisors for graduating. From the start of their training, residents are immersed in medical practice and they mostly learn through feedback and experiential learning. One can imagine that in a situation as complex as residency, both task and relation-oriented leadership behaviours can contribute to residents’ job satisfaction. Residents experience uncertainty and role ambiguity because they transition into novel and unclear tasks, relationships and responsibilities [13–15]. Such uncertainty and role ambiguity may cause a decrease in job satisfaction [16, 17]. The theory of Hersey and Blanchard [12], which was developed for work settings, emphasizes task-oriented leadership behaviours to decrease ambiguity and insecurity, for example by structuring and clarifying the work situation. However, in a medical training situation we also expect relation-oriented leadership behaviours to help in decreasing ambiguity and insecurity, for example by helping residents cope with emotions that emerge from transitions, unclear situations and role ambiguity [10, 16, 18]. In other words, both task and relation-oriented leadership behaviours may increase residents’ job satisfaction and buffer against the stress they experience when encountering new tasks and responsibilities [2, 19, 20].

The aim of our study was to analyze the relation between supervisors’ leadership and residents’ job satisfaction. Our research questions were:

- What is the relation between residents’ appreciation for their supervisor’s leadership in general and job satisfaction?
- What is the relation between residents’ observation of specific leadership behaviours in their supervisors and job satisfaction?

## Methods

### Context, procedure and participants

This study was performed in the context of a large survey on current professional issues and future perspectives of medical graduates (2005–2010) of the University of Groningen, the Netherlands, which started in 2005. The survey and an anonymous return envelope were distributed to the home addresses of graduates who were appointed as residents at the time of this study. They were asked to provide informed consent, complete the questionnaires and send the forms back to the researchers using the return envelopes. In total, 117 (82 %) of the residents provided informed consent and participated in our study.

### Ethical approval

When the survey project started in 2005, no ethical review board was available for medical education research and ethical review is not required by law in The Netherlands. However, the survey study was carried out conform ethical standards in medical education research, by guaranteeing confidentiality, analyzing data anonymously, and ensuring that no possible harm to participants arises from our study [21–23]. Furthermore, participants consented for participation.

### Measurements

#### Job satisfaction

From the literature, we derived 13 aspects that were considered important for physicians job satisfaction [3, 5–8]: (1) opportunities for personal development, (2) professional accomplishments, (3) control over work planning, (4) control over work content, (5) administrative work, (6) cooperation with supporting personnel, (7) cooperation with colleagues, (8) appreciation from supporting personnel, (9) appreciation from colleagues, (10) income, (11) cooperation with management, (12) work-life balance and (13) appreciation from patients. The residents rated their satisfaction with each aspect on a 10-point scale ranging from 1 (not satisfied at all) to 10 (completely satisfied).

#### Factor analysis for grouping aspects of job satisfaction

We performed exploratory factor analysis with Varimax rotation to explore which domains of job satisfaction were covered by the 13 aspects of job satisfaction. To find the best factor solution, we applied three psychometric criteria as described by Schönrock-Adema et al. [24]: (1) point of inflection of the screeplot, (2) eigenvalues >1.5, and (3) minimum percentage of additional explained variance of approximately 5 %. We also took the interpretability of possible factor solutions into account. The best solution we found, based on these criteria, was a four-factor solution. The factors were interpreted as satisfaction with personal growth, satisfaction with autonomy, satisfaction with affective job aspects, and satisfaction with instrumental job aspects. The personal growth factor (Cronbach’s α = 0.63) comprised three aspects of job satisfaction pertaining to professional as well as personal growth: satisfaction with professional accomplishments, satisfaction with personal development, and satisfaction with appreciation from colleagues. The autonomy factor (α = 0.60) included three aspects of job satisfaction: satisfaction with control over work planning, satisfaction with control over work content, and satisfaction with the balance between work and personal life. The affective factor comprised four aspects of job satisfaction (α = 0.71): satisfaction with appreciation from patients, satisfaction with appreciation from supporting personnel, satisfaction with cooperation with colleagues and satisfaction with cooperation with supporting personnel. All aspects in this factor pertained to interpersonal and social relations amongst workers and patients. The instrumental factor (α = 0.60) included three aspects of job satisfaction: satisfaction with cooperation with management, satisfaction with income, and satisfaction with administrative tasks. All aspects in this factor concerned preconditions of the job that usually cannot be easily changed by residents (see Table 1 for the exact factor loadings).

**Table 1:** Factor loadings of the final 3-factor solution of job satisfaction

Aspects	Components			
	1	2	3	4
Affective domain (α .72)				
Appreciation from patients	.440	.303	.302	.015
Appreciation from supporting personnel	.822	.049	-.091	.254
Cooperation with colleagues	.515	.432	.340	.009
Cooperation with supporting personnel	.895	.027	.074	.026
Personal growth domain (α .63)				
Personal development	.206	.730	.153	.140
Professional accomplishments	-.131	.786	.014	-.007
Appreciation from colleagues	.278	.670	.236	.033
Autonomy domain (α .60)				
Control over work planning	.086	.233	.708	-.013
Control over work content	-.020	.159	.816	.181
Balance work-life	.145	.065	.561	-.108
Instrumental domain (α .60)				
Cooperation with management	.169	.059	.104	.779
Income	.098	.123	-.129	.758
Administrative work	-.150	−191	.284	.593

#### Supervisors’ leadership

The residents rated their overall appreciation of their supervisors’ leadership with one item on a 10-point scale, ranging from 1 (no appreciation at all) to 10 (ultimate appreciation). Frequency of observed task and relation-oriented leadership behaviours was measured using four items. For reasons of feasibility, we limited our study to four separate leadership behaviours, derived from the Situational Leadership Theory [12]. We chose these behaviours, because (1) they can be seen as the “basic” leadership behaviours every physician needs to learn and (2) they can easily be observed in daily practice. The items had to be scored on a 4-point scale ranging from 1 (never observed) to 4 (observed very often). Two items addressed task-oriented leadership behaviours: “The supervisor tells me exactly how, where, and when to perform tasks” (*specific instructions*) and “The supervisor gives general directions to complete work” (*global instructions*). The other two items addressed relation-oriented leadership behaviours: “There is a two-way communication with the supervisor” (*two*-*way communication*), and “The supervisors makes mutual decisions with the resident” (*mutual decision*-*making*) [12]. We asked the residents to take the supervisor in mind with whom they worked most regularly at that time and to fill out the statements according to their experiences with this specific supervisor.

#### Statistical analysis

The independent variables of the study were *overall appreciation* of supervisor leadership and the four specific leadership behaviours: *specific instructions*, *global instructions*, *two*-*way communication* and *mutual decision*-*making*. The dependent variables of our study were the four job satisfaction factors: personal growth, autonomy, affective, and instrumental. We calculated the means for all independent and dependent variables, the correlations between them –high correlations among pairs of predictor variables are indicative of multicollinearity– and the VIF (Variance Inflation Factor). VIF values above 4 indicate that the beta values may be overestimated [25].

After testing for multicollinearity, we performed linear regression analyses to investigate the relation between leadership and job satisfaction. Considering the results of the factor analysis, we decided to perform four regression analyses, one for each job satisfaction factor, to examine the influence of *overall appreciation* and observation of the specific leadership behaviours. To distribute the weight of the separate variables evenly over the scales, we calculated z-scores for each variable before summing the scores into the four scales. We added the specific leadership behaviours separately to the regression models as independent variables. In all regression analyses, we controlled for gender to correct for possible gender differences in the way supervisor leadership is perceived. Additionally, we controlled for the number of years in residency training because experience may lead to a decrease in role ambiguity and insecurity and, therefore, influence job satisfaction outcomes [16, 17].

## Results

### Descriptives

The gender distribution, 37 males and 63 % females, was representative of the resident population. Residents were highly satisfied with their opportunities for personal development (*M* = 8.2, *SD* = 0.67), professional accomplishments (*M* = 8.1, *SD* = 0.79), and cooperation with colleagues (*M* = 8.0, *SD* = 0.65). They were least satisfied with work content (*M* = 6.9, *SD* = 0.84), administrative work (*M* = 5.9, *SD* = 1.4), cooperation with management (*M* = 6.9, *SD* = 1.0) and income (*M* = 6.9, *SD* = 1.2), which were all rated below 7. Residents rated the remaining 6 aspects of job satisfaction between 7 and 8. On average, overall appreciation for supervisors’ leadership was 7.4 (*SD* = 1.03).

The correlations between all variables are displayed in Table 2. We found significant positive correlations between *overall appreciation* and specific leadership behaviours (see Table 2), but the VIF (Variance Inflation Factor) values were all below 4, indicating that multicollinearity would not influence the results severely.

**Table 2:** Overview of correlations between variables in the study

Variables	M ± SD	1	2	3	4	5	6	7	8	9
Appreciation leadership (1 = no appreciation,10 = ultimate appreciation)										
1. Overall appreciation	7.4 ± 1.0	-	.115	.264*	.485*	.380*	.265*	.249*	.081	.218*
Leadership behaviours (1 = never, 4 = often)										
2. Specific instructions	2.7 ± .80		-	.370*	.044	.294*	.105	.068	.024	.192
3. Global instructions	3.1 ± .67			-	.456*	.455*	-.053	-.075	-.020	.088
4. Two-way communication	3.0 ± .94				-	.711*	.154	.159	.051	.044
5. Mutual decision-making	2.7 ± .83					-	-.033	.083	.032	.145
Job satisfaction domains (1 = not satisfied 10 = completely satisfied)										
6. Affective domain job satisfaction	7.8 ± .55						-	.436*	.264*	.170
7. Personal growth domain	8.1 ± .51							-	.354*	.088
8. Autonomy domain job satisfaction	7.1 ± .68								-	.245*
9. Instrumental domain job satisfaction	6.6 ± .93									-

^*^ *p* < 0,05

### Regression analyses

The regression models, with overall appreciation and the specific leadership behaviours as independent variables, significantly predicted the variance in the affective (*p* = 0.012) and instrumental (*p* = 0.009) job satisfaction scales (14 and 13 %, respectively). Overall appreciation of supervisor leadership as well as the degree to which residents observed *specific instructions* (task-oriented) and *two*-*way communication* (relation-oriented) in their supervisors positively influenced the affective job satisfaction scale scores (β = 0.792, *p* = 0.017; β = 0.975, *p* = 0.008; and β = 1.625, *p* = 0.008, respectively). The more often residents observed *mutual decision*-*making* (relation-oriented), the lower they scored on the affective job satisfaction scale (β = 1.285, *p* = 0.007). The significance of the regression model for the instrumental job satisfaction scale was attributable to gender, a control variable (β = 2.320, *p* = 0.001). Women scored higher on the instrumental job satisfaction scale than men (see Table 3 for regression models).

**Table 3:** Results of the four regression analyses, per domain of job satisfaction

Variables	Affective domain			Personal growth domain			Autonomy domain			Instrumental domain		
	β	SE	p	β	SE	P	β	SE	P	β	SE	P
Intercept	498.06	453.02	.275	162.12	364.57	.658	883.57	358.54	.016	10.47	.354.37	.997
Overall appreciation	.792	.324	.017*	.524	.261	.048	.066	.265	.804	.393	.248	.118
Specific instructions	.975	.357	.008*	.384	.288	.185	.440	.283	.123	.208	.271	.446
Global instructions	-.693	.374	.067	-.587	.301	0.54	-.318	.309	.06	-.208	.289	.475
Two-way communication	1.376	.502	.008*	.487	.404	.232	.576	.422	.177	-.631	.392	.112
Mutual decision-making	−1.285	.678	.007*	-.154	.376	.684	-.319	.381	.404	.523	.378	.170
Gender	-.906	.678	.185	-.388	.546	.537	-.257	.538	.634	1.864	.560	.001*
Years in training	-.250	.225	.270	-.082	-.049	.651	-.440	.178	.016	-.008	.176	.963

^*^ *P* < .05

## Discussion

Overall appreciation of supervisors’ leadership positively influenced residents’ satisfaction with the affective aspects of their jobs. Similarly, the more residents observed *specific instructions* and *two*-*way communication* in their supervisors, the higher their satisfaction with the affective aspects of their jobs was. In contrast, frequent observation of *mutual decision*-*making* was related to less satisfaction with the affective job aspects.

The relation we found between appreciation for supervisor leadership and resident job satisfaction is congruent with the literature from outside the medical field [9]. In general, organizational literature emphasizes the positive effect of supportive leadership on job satisfaction [10, 16, 18]. In line with the literature, we defined job satisfaction as a multifaceted concept. We found four job satisfaction factors to be important for residents. These factors are in line with the three domains distinguished in Ostroff’s taxonomy of organizational climate perceptions [26]: the cognitive, affective and instrumental aspects of the organizational climate. Our first two factors –personal growth and autonomy– fit within the cognitive domain and the other two factors clearly correspond with the affective and instrumental domains identified by Ostroff. We found significant correlations between *overall appreciation* and job satisfaction in the affective, personal growth and autonomy domain. However, after controlling for number of years in training and gender, we only found an effect of leadership in the affective domain of job satisfaction. This domain covers the perceived appreciation of and collaboration with colleagues.

An explanation for the influence of supervisor leadership on resident job satisfaction may be found in the nature of the job. Residents experience all sorts of insecurities and role ambiguity [10, 16, 18]. *Specific instructions* may provide structure to residents’ jobs and help them deal with complex situations which, in turn, may increase appreciation for and collaboration with other colleagues. *Two*-*way communication* may offer support and help residents deal with their insecurities. As a result, residents feel more satisfied with how they work with others. To stimulate this process, it is important that supervisors display these leadership behaviours. Furthermore, supervisors serve as role models for residents [27]. Supervisor leadership may be mimicked by residents in interaction with their colleagues. These colleagues, in turn, may appreciate the residents’ behaviours which may help residents establish a good working relationship and increase their job satisfaction in the affective domain.

The more often residents observed *mutual decision*-*making* in their supervisors, the less satisfied they were with the affective aspects of their jobs. A possible explanation for this finding is that mutual decision-making may only be applicable within the residents’ zone of proximal development [28]. More specifically, mutual decision-making may be an optimal strategy when the resident has sufficient expertise to weigh a situation, but lacks the expertise to fulfill a task autonomously. In such situations, mutual decision-making may be empowering and, therefore, increase job satisfaction [29]. When residents have too little or too much expertise, however, mutual decision-making may have counterproductive effects on job satisfaction. Residents who still find it difficult to assess the task at hand may feel unequipped for the responsibility that comes along with mutual decision-making. Residents who feel capable of acting autonomously in a situation, on the other hand, may not benefit from mutual decision-making because it interferes with their work-related autonomy. Further research is needed to gain insight in the circumstances under which specific leadership behaviours such as mutual decision-making are effective.

We did not find any relations between the instrumental domain of job satisfaction and supervisor leadership. The aspects in this domain, for example satisfaction with income, administrative tasks and working together with management, can be seen as the “hygiene” factors of the job. Hygiene factors do not positively influence job satisfaction when they are present and evaluated, however, they will negatively influence job satisfaction when they are absent or employees are unsatisfied with these factors [30]. For example, residents’ job satisfaction may be negatively influenced by administrative load. Although residents were least satisfied with the instrumental aspects of their jobs compared to the other job domains, they still positively evaluated these aspects. Therefore, supervisor leadership may not contribute to job satisfaction in this domain.

The fact that supervisor leadership influences resident job satisfaction has some practical consequences. Leadership training may be helpful in creating more awareness among supervisors about the way their leadership behaviours influence residents’ job satisfaction. Based on our findings, it seems important that supervisors realize which behaviours are beneficial for residents’ wellbeing. Leadership training may support supervisors in making residents feel and perform better [31].

In the design of this study, we limited ourselves to questions about SLT leadership behaviours derived from a transactional leadership theory [12]. The rationale behind this choice was that transactional leadership behaviours can easily be observed. The limitation to transactional leadership behaviours may have been the reason for not finding a relation between leadership and job satisfaction in the autonomy and personal growth domains. Satisfaction with these domains may be more sensitive to transformational leadership behaviours of a supervisor. Transformational behaviours, such as creating a vision and enhancing creative thinking among employees, may inspire residents to think about their own personal and professional development. Studies from outside the medical field have already shown that transformational leadership behaviours are important for job satisfaction [29, 32]. Future research should focus on the influence of transformational leadership on job satisfaction in a residency training setting.

This study was limited to residents’ job satisfaction and perceptions of their supervisor’s leadership as measured by survey items we designed ourselves. These items were not validated through a pilot study. However, all survey items were selected based on literature and face validity and either directly related to aspects or behaviours described in literature (the leadership behaviours) or their content validity (job satisfaction aspects) was supported by the outcomes of the exploratory factor analysis that we performed. The job satisfaction factors found could clearly be related to Ostroff’s taxonomy of organizational climate [26], a theoretical framework that was identified by Carr et al. as representing the incorporation of the existing literature [33]. Another limitation of this study may be the fact that we based our study on residents’ perceptions. It could be argued that direct observation of supervisor leadership behavior might have offered a more objective measure of supervisors’ leadership behaviour, because perceptions may not always reflect reality. However, in this study the use of residents’ perceptions was inevitable, because both the way leadership is experienced and job satisfaction involve internal processes that happen within individuals’ minds and, as a consequence, cannot be assessed objectively [34]. Furthermore, questions could be raised about which supervisor a resident had in mind while filling out the questionnaire, as residents have to deal with multiple supervisors during their training. We tried to minimize this problem by asking the residents to keep the supervisor in mind with whom they worked most regularly at that time. Future research investigating dyads of residents and supervisors in clinical practice may increase our understanding of the dynamics of supervisor leadership and resident job satisfaction.

## Conclusion

In conclusion, supervisor leadership behaviours influenced residents’ satisfaction with the affective aspects of their jobs. Observation of the supervisor leadership behaviours *specific instructions* and *two*-*way communication* positively influenced residents’ satisfaction with the affective aspects of their jobs, whereas observation of *mutual decision*-*making* negatively influenced job satisfaction in the affective domain. Based on our findings we recommend that supervisors become more aware of whether and how their leadership influences residents’ job satisfaction.
